# Attitudes and practices for antibiotic prescription and antimicrobial resistance among general physicians -Findings from a multi-country survey

**DOI:** 10.1371/journal.pgph.0004558

**Published:** 2025-05-07

**Authors:** Nitin Maksane, Karen Langfeld, J.P. Bhaskar, Sanchayita Sadhu, James van Hasselt

**Affiliations:** 1 Global Medical Affairs, Mumbai, India; 2 Global Medical Affairs, Brentford, London, United Kingdom; 3 Global Medical & Clinical Affairs, Mumbai, India; 4 Development Biostatistics, Bengaluru, India; 5 Regional Medical Affairs, Gauteng, South Africa; University of Oslo Faculty of Medicine: Universitetet i Oslo Det medisinske fakultet, NORWAY

## Abstract

This study aimed to assess the attitudes, and practices (AP) of general physicians (GPs) regarding antibiotic prescribing and antimicrobial resistance (AMR). A cross-sectional, descriptive AP study was conducted by surveying GPs treating community acquired respiratory tract infections (RTIs) across nine countries, including India, Pakistan, Algeria, Thailand, Vietnam, Egypt, Morocco, the United Arab Emirates, and Saudi Arabia. A 29-item, web-based questionnaire was used to collect data between October-2023 and December-2023. Overall, 9249/14207 invited GPs responded, and 1008 responses were included in the analysis after quality control (3341 terminated due to eligibility, 4764 dropped out without completion, 136 excluded for quality concern). Of the included respondents, 78.8% were male and 98% were aged ≥35 years. 41% of GPs agreed, 33% disagreed, and 27% were neutral to questions regarding whether or not antibiotics are helpful in treating infectious respiratory diseases. In total, 62% of GPs agreed that AMR is a concern in their country and 63% agreed that prescribing antibiotics in primary care results in AMR. Pregnant women and patients with comorbidities were populations for whom selecting an appropriate antibiotic was most challenging; 38% of GPs found prescribing antibiotics to children was difficult. Difficulty in correlating susceptibility data (53%), limited availability of information on antibiotics (52%), and lack of availability of appropriate antibiotics (51%) were important challenges for appropriate antibiotic selection. Overall, 94% of GPs agreed that there is a need for frequent training on antibiotic therapy, with 33% and 49% recommending quarterly and biannual trainings, respectively. This study identified current practices, and possible gaps in appropriate antibiotic prescribing for RTIs. As an outcome, specific training needs could be identified to assist GPs with appropriate antibiotic prescribing in an outpatient setting.

## Introduction

When used appropriately, antibiotics can be lifesaving; however, excessive, and inappropriate use of antimicrobials can lead to issues such as antimicrobial resistance (AMR). AMR has become a serious global public health concern, contributing to longer hospital stays and millions of deaths each year [1]. A systematic literature review conducted by the Antimicrobial Resistance Collaborators estimated that, in 2019, bacterial AMR contributed to 4.95 million deaths and was directly responsible for 1.27 million deaths globally [1]. According to the systematic analysis conducted by Naghavi et al. by 2050, approximately 1.91 million deaths could be attributable to AMR, while 8.22 million deaths could be associated with AMR [2]. It is estimated that up to as many as 40–60% of all the infections are caused by resistant microorganisms [3].

In addition to its impact on global health, AMR has a significant health economic impact. Overprescribing and misuse of antibiotics are the primary causes of increasing antibiotic resistance [4]. During the period between 2000 and 2015, the global per capita consumption of Watch antibiotics increased by approximately 91%, while the consumption of Access antibiotics increased by 26.2% [5].

Another study demonstrated that between 2000 and 2015, antibiotic use was estimated to have increased by 65% in 76 countries [6]. Consequent to the increased rate of prescription, the costs for infection control are also increasing. In a systematic literature review conducted by Poudel et al, the per patient per episode cost for resistant infections was as high as US$29,289, with a 1.844 odds ratio for mortality [7].

Furthermore, between 2000 and 2018, antibiotic prescribing in the paediatric population was shown to have increased by 45%, with a specific increase in the use of broad-spectrum antibiotics during the same timeframe [8]. In a study conducted in three low- to middle-income countries (LMICs), up to 76% of antibiotic prescriptions in paediatric patients were determined to be inappropriate [9]. Apart from inappropriate prescribing, erroneous prescriptions were identified as another concern, where either an incorrect dose was prescribed or the antibiotic was prescribed for an incorrect duration [10]. It is noteworthy that 80%–90% of antibiotic prescriptions are made by general practitioners (GPs) in outpatient care, approximately 50% of which are estimated to be inappropriate [11–13]. Community-acquired respiratory tract infections (CA-RTIs) are understood to be generally mild self-limited illnesses and constitute the major reason for antibiotic prescription [14–16]. Lack of adequate guidelines and training on appropriate antibiotic use, shortage of diagnostic microbiology laboratories, lack of adequate time with patient for appropriate diagnosis and patients’ expectations of antibiotic prescription have been identified as major challenges impacting appropriate antibiotic prescribing [9,17,18].

Although the main factors responsible for emerging AMR are known, effective interventions to address the issue are still required. GPs often act as first line of defense in most health systems regarding management of CA-RTIs and antibiotic prescription. Hence, supporting GPs to achieve appropriate antibiotic prescribing is a valid contribution to counter rising AMR in LMICs. Therefore, it is important to understand the existing practices followed by the GPs regarding AMR, particularly in LMICs, and their attitudes toward appropriate antibiotic prescribing. With this objective, we aimed to conduct a comprehensive questionnaire-based attitudes, and practices (AP) survey to understand antibiotic prescribing behaviour of GPs across nine countries in Asia, Africa, and the Middle East. Although there are similar studies in individual countries, to the best of our knowledge no single study has objectively evaluated the attitudes and practices of GPs in such a diverse Low-middle income geographical area, with a lesser focus on prevalent practices. The study’s findings could pinpoint deficiencies in the current primary care system and generate actionable insights to ensure appropriate antibiotic prescriptions, thereby reducing antimicrobial resistance.

## Methods

### Study design

A descriptive cross-sectional survey was conducted from 23^rd^ October 2023–27^th^ December 2023 with a target to include at least 1000 GPs across nine countries, including India, Pakistan, Algeria, Thailand, Vietnam, Egypt, Morocco, the United Arab Emirates, and Saudi Arabia. The primary objective was to assess the attitudes and practices of GPs regarding antibiotic use in patients with CA-RTIs and the increasing AMR.

The study was approved by the Royal Pune Independent Ethics Committee (registration no. ECR/45/Indt/MH/2013/RR-19; approval no. RPIEC091023) in India.

### Development of the questionnaire

A 29-item, web-based questionnaire was designed following a comprehensive literature search tailored to the study objectives [19,20]. Additional questions were included to collect demographic information of the study participants. The questionnaire was developed using best practices for survey instrument development [21]. The questionnaire was developed with the assumption that upper respiratory tract infections are more frequently encountered RTIs in the primary care [22,23], however, questions also considered the commonly encountered lower RTIs such as community-acquired pneumonia and acute exacerbation of chronic obstructive pulmonary disease (COPD). Although the focus of the survey was to understand the attitudes and practices of the GPs, a few questions assessing the baseline knowledge of the GPs were included in the study.

After development of the questionnaire and before study initiation, the questionnaire was validated by a group of ten experts comprising GPs and specialists. The content validity index (CVI) for each question was calculated by dividing the number of GPs indicating ‘agree’ or ‘strongly agree’ for the item by the total number of GPs who had rated the question. Average CVIs were calculated to determine the content validity index for the questionnaire. The minimum CVI value for instrument validation was 0.80.

The validated questionnaire was translated into French and Vietnamese for use in Francophone countries and Vietnam, respectively. Backtranslations of the translated versions were validated.

### Study population and sample size

Participating countries were selected based on a feasibility study conducted internally by the sponsor. According to the feasibility study, the included countries had a high antibiotic prescription rate, with majority of the prescriptions made by GPs.

Potential responders were recruited through random selection using a trusted third-party vendor in the selected countries for the study. GPs were included if they were practicing in the country of interest, had at least 5 years of experience, and had treated a minimum of 20 adult or paediatric patients per month suffering from CA-RTIs. Written informed consent was obtained from each GP before survey participation using the same online link as the survey. The survey launched only if the participant provided consent, and the consent was recorded in the data file.

The sample size was calculated using a web-based calculator—Raosoft Web. Given the general descriptive and non-inferential nature of the study design, the sample size was based on logistical considerations to provide reasonable estimates for the primary and secondary outcomes. Considering a 5% margin of error and using 95% confidence interval, a sample size of 1000 participants was calculated to be appropriate for the study. Table A in S1 Table shows the range of margin of error expected (at a 95% confidence interval) for various levels (50%–90%) of response distribution in percentage values by GPs for a sample size between 700 and 1200 responders.

### Online survey distribution and data collection

Data were collected between October 2023 and December 2023. Respondents who met the eligibility criteria were invited to participate in the survey. The developed survey questionnaire (in English, French, and Vietnamese) was converted to an online version using the software platform Forsta/Decipher (Version 3.01) and circulated among participants per their preferred language using participant-specific unique links. All participants were blinded to the sponsor’s identity. The identifiable data collected for participating GPs were pseudo-anonymised. An honorarium, which was aligned with the local fair market value and as per applicable local jurisdiction, was paid to the participants.

All the collected data were checked for completeness and validity. Incomplete responses were identified and excluded from the analysis.

### Data analysis

Data were collected using pre-determined Microsoft Excel sheets. Statistical analyses were primarily explorative and descriptive in nature and were performed using Quantum, SPSS, and R software (Version 9.3.1 or higher). Demographic data are presented as numbers and percentages and quantitative responses are presented as tables and graphs. The ranking questions were analysed by calculating the average rank of each item as follows:

W × Y (where W is the weight of the ranked position and Y is the response count for answer choice).

The application of weights works in reverse, where the most preferred item (#1) is assigned the highest weight, and the least preferred item is assigned the lowest weight. The weight count depends on the total number of items in the question.

## Results

### Demographics and participant characteristics

A total of 14207 survey invitations were sent to GPs, 9249 of whom responded (response rate: 65.1%). Of these, 3341 were terminated as a result of eligibility criteria cut-off and a further 4764 dropped out without completion. Hence, 1144 responses were considered for quality check. After quality control of the anonymised data and reconciliation of incomplete responses, 1008 responses were included in the analysis. Of the 1008 participants, 78.8% were male and the age ranged between 28 and 61 years, with 90.8% aged between 35 and 54 years. The highest educational qualification was MBBS for 81.7% of GPs, and the median duration of practice was approximately 14 years (range: 5–35 years). Most GPs were practicing in private institutions, including private hospitals (43.6%) and outpatient facilities (19.2%), followed by government institutions (26%). Approximately 60% of the respondents were from urban areas, 18% were from semi-urban areas, and 15.4% were from semi-rural areas. The median number of patients seen in a month was 350 adults (range: 90–600) and 100 children (range: 15–300). Detailed demographics of the participants are summarised in Table 1.

**Table 1:** Demographics and characteristics of survey respondents.

	Respondents (N = 1008), n (%)
Age group^*^, years	
25–34	17 (1.7)
35–44	490 (48.6)
45–54	425 (42.5)
≥55	76 (7.5)
**Sex** ^*^	
Male	794 (78.8)
Female	214 (21.2)
**Country** ^*^	
India	163 (16.2)
Pakistan	100 (9.9)
Algeria	120 (11.9)
Vietnam	150 (14.9)
Morocco	100 (9.9)
Saudi Arabia	101 (10.0)
Egypt	72 (7.1)
UAE	120 (11.9)
Thailand	82 (8.1)
**Highest qualification**	
MBBS	824 (81.7)
Diploma (after MBBS)	184 (18.3)
**Physician practice setting** ^*^	
Government hospital	188 (18.7)
Government OPD (outpatient clinic)	74 (7.3)
Private hospital	439 (43.6)
Private outpatient facility/consulting room (outpatient clinic)	194 (19.3)
Primary health care clinic (outpatient)	113 (11.2)
**Type of geographical area/location of physician practice** ^*^	
Rural	62 (6.2)
Semi-rural	155 (15.4)
Urban	610 (60.5)
Semi-urban	181 (17.9)
**Payment mode at clinic/hospital** ^*^	
Private insurance	645
Public insurance	387
Out-of-pocket payment	833
**Duration of medical practice (years)**	
Mean	15.4
SD	6.91
Median	14
Minimum	5
Maximum	35
**Consultation for adult patients (n per month)**	
Mean	353.3
SD	95.27
Median	350
Minimum (n)	90
Maximum (n)	600
**Consultation for paediatric patients (n per month)**	
Mean	107.9
SD	59.58
Median	100
Minimum (n)	15
Maximum (n)	300

*Data are presented as number (n) of respondents. OPD, outpatient department; SD, standard deviation; UAE, United Arab Emirates.

### Baseline knowledge of GPs regarding antibiotic prescription

Table B in S1 Table provides an overview of all the responses pertaining to questions related to knowledge of GPs regarding antibiotic prescription.

**Fig 1:**
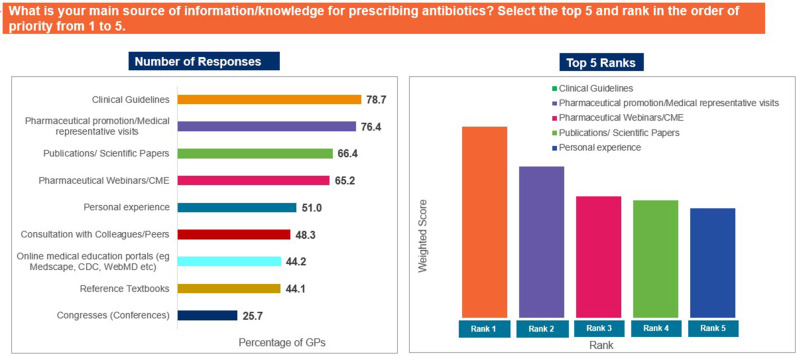
Source of information for antibiotic prescribers. Abbreviations: CDC, Centers for Disease Control; CME, Continued Medical Education; GP, general physician; URTI, upper respiratory tract infection.

**Fig 2:**
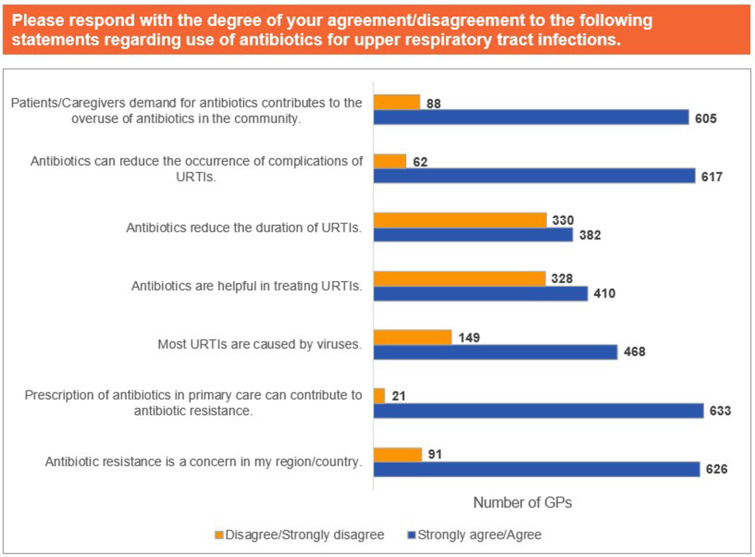
Use of antibiotics for URTIs. Abbreviations: URTI, upper respiratory tract infection.

Clinical guidelines (78.7%), information shared by medical representatives (76.4%), and pharmaceutical webinars (65.2%) were identified as the most valuable sources of information regarding antibiotic prescription (Fig 1). Further, 62.1% of the participants agreed that AMR is a concern in their country of practice, while 62.8% agreed that prescription of antibiotics in primary care contributes to AMR; 46.4% of GPs concurred that most upper respiratory tract infections (URTIs) are caused by viruses. Moreover, 40.7% of GPs agreed, while 32.5% did not agree that antibiotics are helpful in treating URTIs (Fig 2).

### Attitudes and practices of GPs regarding antibiotic prescription

An overview of all responses regarding attitudes and practices is summarised in Table C and Table D in S1 Table, respectively.

### Challenges in appropriate antibiotic prescribing

**Fig 3:**
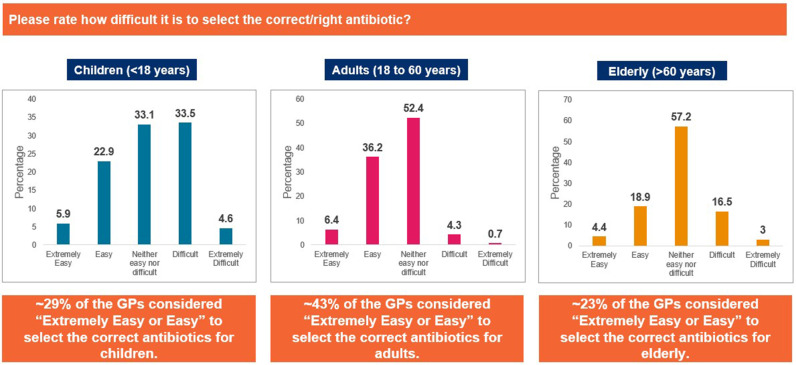
Difficulty in appropriate antibiotic prescription. Abbreviations: GP, general physician.

**Fig 4:**
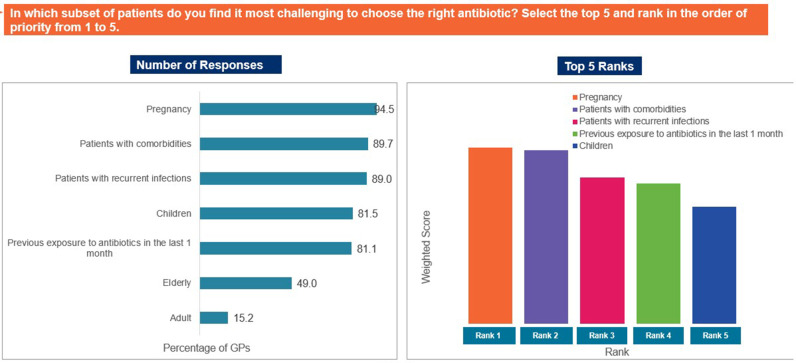
Challenging populations for appropriate antibiotic prescription. Abbreviations: GP, general physician.

**Fig 5:**
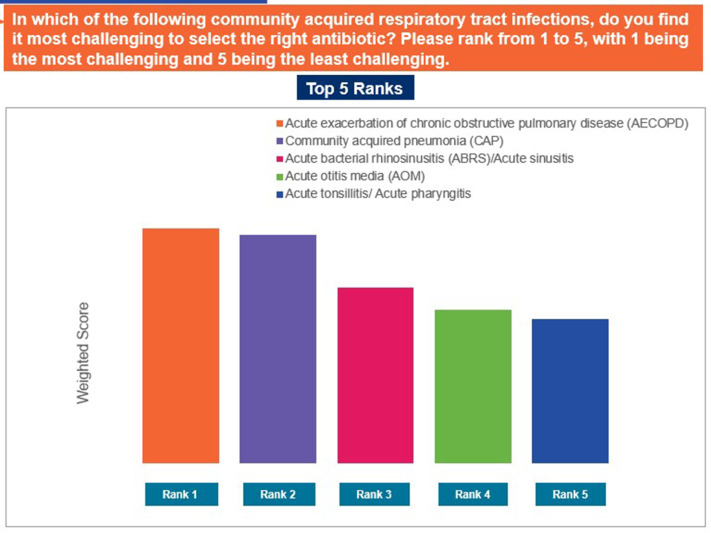
Challenging infection for appropriate antibiotic prescription. Abbreviations: GP, general physician.

**Fig 6:**
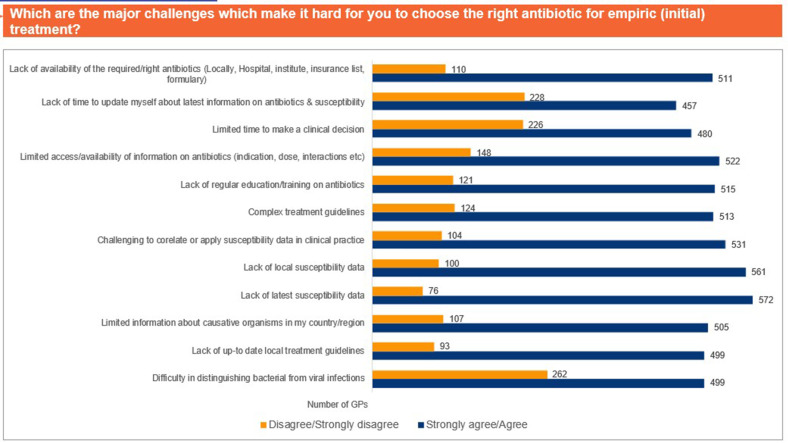
Major challenges for appropriate antibiotic selection. Abbreviations: GP, general physician.

Approximately 38% of the participants rated that they found prescribing antibiotics to children difficult (33.5%) to extremely difficult (4.6%), whereas majority of the GPs found prescribing antibiotics to adults (52.4%) and the elderly (57.2%) neither easy nor difficult (Fig 3). Pregnant women (94.5%) and patients with comorbidities (89.7%) were identified as populations for whom selecting the right antibiotic was the most challenging (Fig 4). Acute exacerbation of chronic obstructive pulmonary disease, community-acquired pneumonia, and acute bacterial rhinosinusitis were identified as the most challenging CA-RTIs for which to prescribe the correct antibiotic (Fig 5). Approximately 57% of the participants agreed that a lack of the latest susceptibility data made it difficult for them to prescribe appropriate antibiotics. Difficulty in correlating the susceptibility data (53% agreed), limited availability of information on antibiotics (52% agreed), and lack of availability of appropriate antibiotics (51% agreed) were other important challenges that made it difficult to select an appropriate antibiotic (Fig 6).

### Factors driving AMR

**Fig 7:**
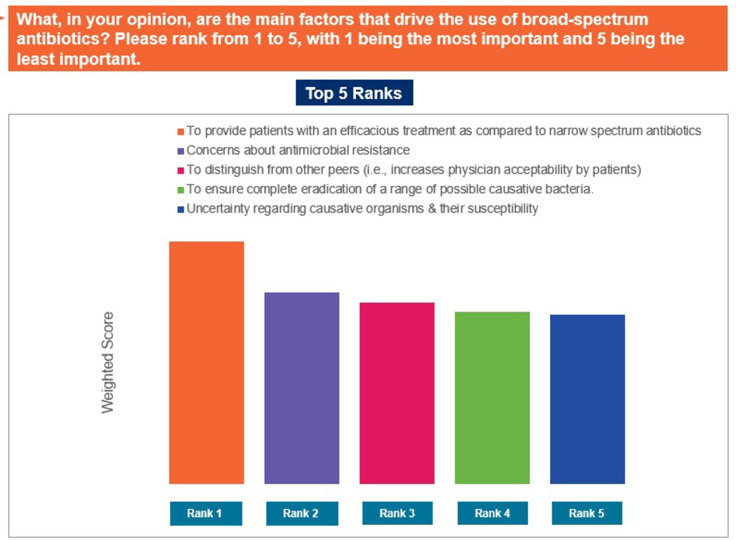
Factors driving use of broad spectrum antibiotics. Abbreviations: GP, general physician.

**Fig 8:**
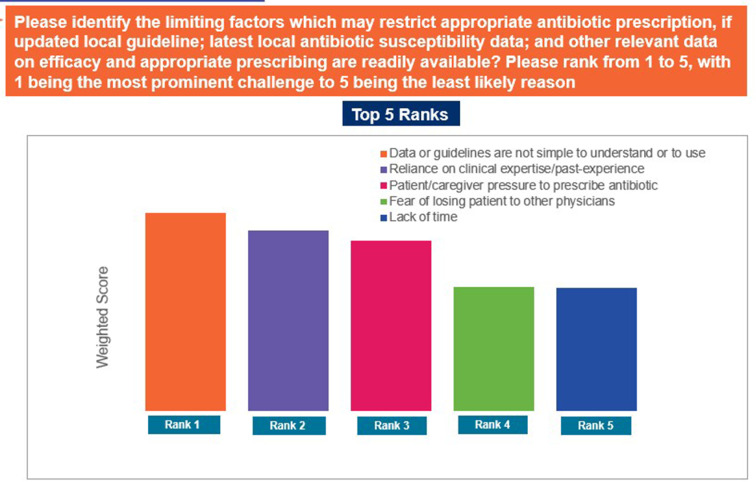
limiting factors for appropriate antibiotic prescription. Abbreviations: GP, general physician.

**Fig 9:**
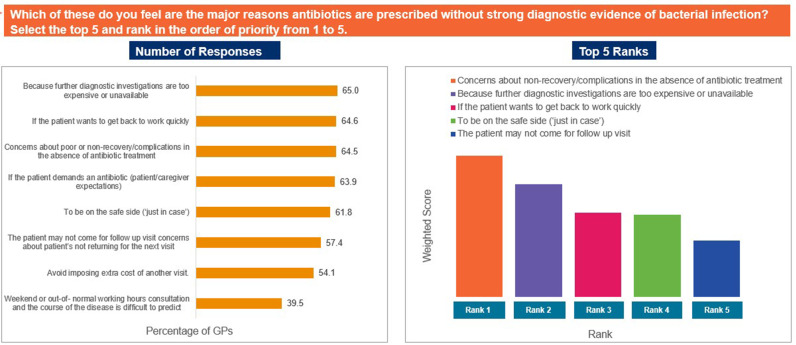
Reasons for antibiotic prescription without stron diagnostic evidence. Abbreviations: GP, general physician.

**Fig 10:**
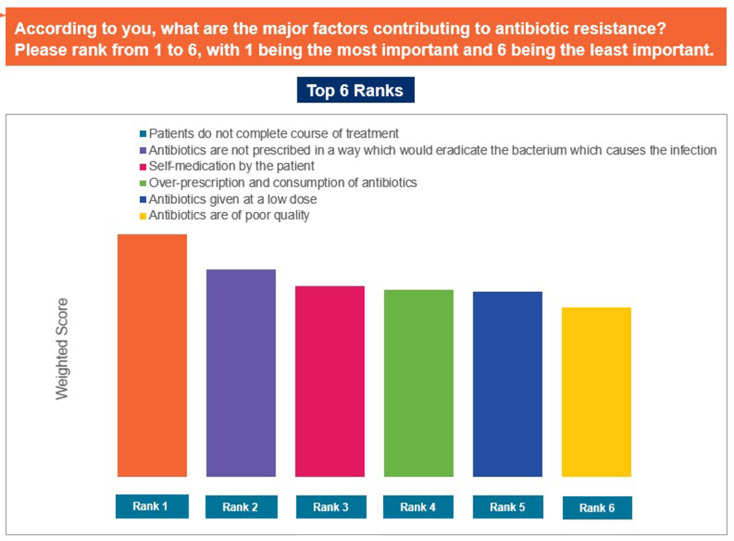
major factors contributing to antibiotic resistance. Abbreviations: GP, general physician.

**Fig 11:**
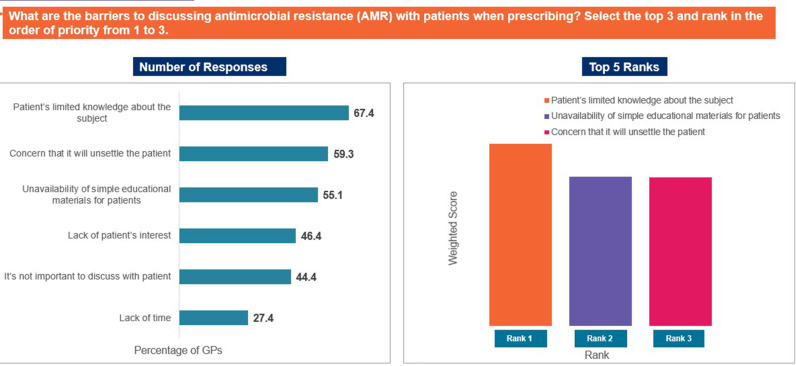
Barriers to discussing AMR with patients. Abbreviations: AMR, antimicrobial resistance; GP, general physician.

The responses ‘to provide patients with an efficacious treatment as compared to narrow spectrum antibiotics’, ‘concerns about AMR’, and ‘to increase practitioner acceptability’ were selected as the top reasons driving use of broad-spectrum antibiotics in the individual’s practices (Fig 7). In contrast, ‘data and/or guidelines are not simple to understand or to use’, ‘reliance on clinical expertise/past-experience’, and ‘patient/caregiver pressure to prescribe antibiotic’ were identified as the top limiting factors that may restrict appropriate antibiotic prescription (Fig 8). The responses ‘concerns about poor or non-recovery/complications in the absence of antibiotic treatment’, ‘because further diagnostic investigations are too expensive or unavailable’, and ‘if the patient wants to get back to work quickly’ were ranked as the major reasons for prescribing antibiotics despite the absence of strong diagnostic evidence (Fig 9). Patients not completing the antibiotic course, improper prescription of antibiotics, and self-medication by patients were identified as the top-ranked factors contributing to AMR (Fig 10). Patients’ limited knowledge, unavailability of simple educational material, and concerns that the information will unsettle the patient were identified as the top barriers limiting discussions with patients regarding AMR (Fig 11).

### Educational needs/training requirements to support appropriate antibiotic prescription and help reduce AMR

**Fig 12:**
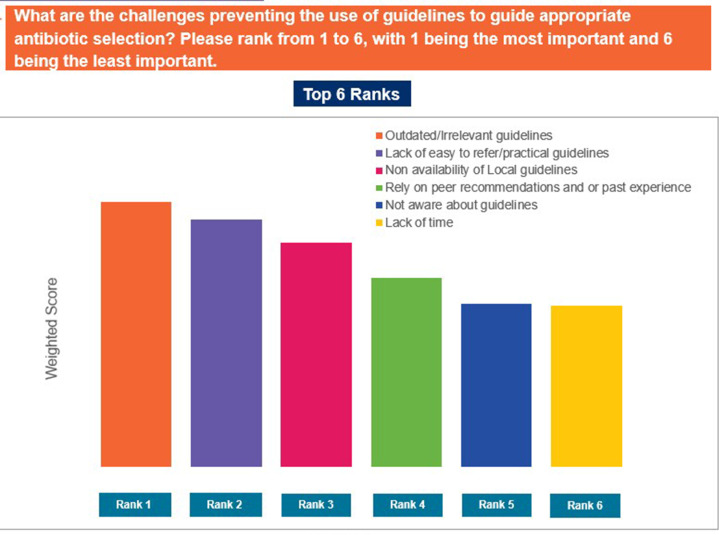
Challenges preventing use of guidelines.

**Fig 13:**
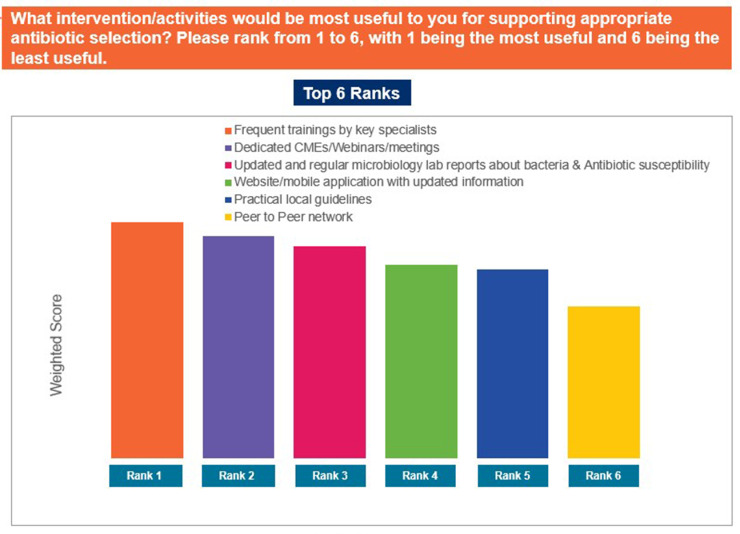
Important activities to promote appropriate antibiotic prescription. Abbreviations: AMR, antimicrobial resistance; CME, Continued Medical Education.

**Fig 14:**
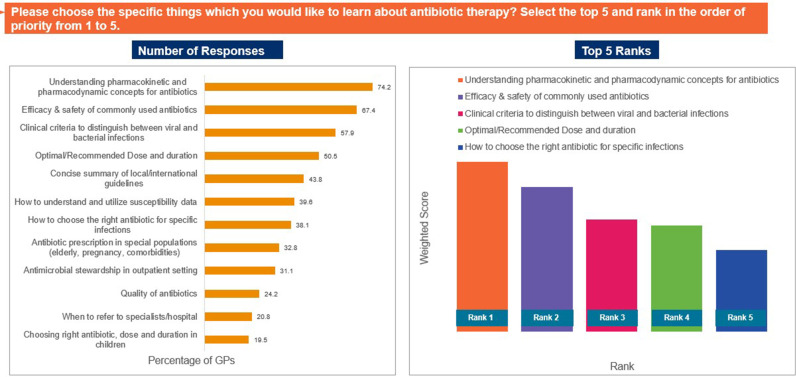
Training requirements to promote appropriate antibiotic prescription. Abbreviations: GP, general physician.

**Fig 15:**
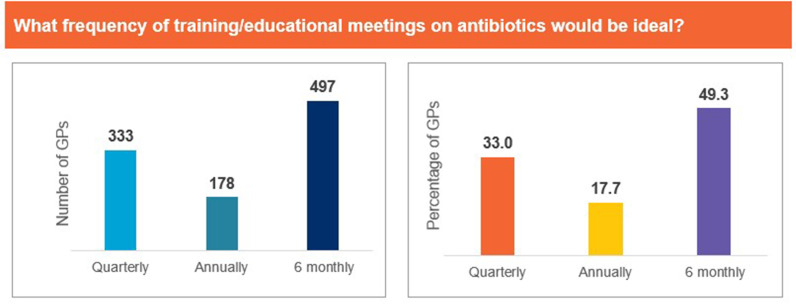
Frequency of trainings to promote appropriate antibiotic prescription. Abbreviations: GP, general physician.

Approximately 94% of the participants agreed that there is a need for training programmes on antibiotic therapy. When asked if they referred to guidelines for appropriate antibiotic selection, 45% of GPs responded that they use guidelines ‘often’, while 32% responded that they use guidelines ‘sometimes’. Outdated guidelines, lack of easy-to-read reference materials, and lack of local guidelines were identified as the top factors limiting the use of guidelines (Fig 12). ‘Frequent training’ and ‘dedicated CME (continued medical education), webinars or meetings’ were identified as the most useful resources for supporting appropriate antibiotic selection (Fig 13). Furthermore, pharmacokinetic/pharmacodynamic properties of antibiotics, efficacy and safety, and clinical criteria to distinguish between bacterial and viral infections were identified as the top topics of interest to responding GPs (Fig 14). Nearly 74% of the GPs noted that they would like to use quick reference materials in addition to educational training. Local physician association portals, local websites, and physician social platforms were identified as the most preferred media to access educational content. Approximately 33% and 49% of GPs believed that training on antibiotics should be conducted on a quarterly basis and biannual basis, respectively (Fig 15). Further, 41% of the participants identified a preference for digital or online meetings, while nearly 32% preferred in-person meetings for participating in educational and training programmes.

## Discussion

This study provides comprehensive information regarding the attitudes and practices of GPs concerning appropriate antibiotic prescribing and AMR in various regions, including LMICs. The participating GPs identified multiple challenges hindering appropriate antibiotic prescribing, factors driving antibiotic overuse resulting in AMR, gaps in available knowledge, and training requirements to assist appropriate antibiotic prescribing in patients with CA-RTIs.

Antibiotic overuse and inappropriate antibiotic prescribing practices are a few of the major causes of AMR [9,10]. The survey identified a few reasons which may be unique for LMICs which result in AMR. According to the survey results, GPs face a significant challenge in prescribing appropriate antibiotics to vulnerable patient populations, such as children, the elderly, pregnant women, and patients with comorbidities. This challenge is further accentuated by factors such as lack of susceptibility data and difficulty in correlating these data, when available. Furthermore, the lack of susceptibility data increases the use of broad-spectrum antibiotics, which may lead to increased AMR. Lack of infrastructure, lack of adequate surveillance, and weak laboratory capacity are some of the barriers to acquiring the necessary data for appropriate antibiotic prescription [24]. Cost may also be a major reason for not requesting microbiological susceptibility testing to guide appropriate prescribing, as most LMICs have self-funded healthcare systems [25]. It is also important to note that while the majority of the CA-RTIs are viral in origin and are self-limiting, a large proportion of these infections are treated with antibiotics [26]. Furthermore, Iskander et al. note limited staffing and their training, lack of appropriate communication amongst laboratory staff and GPs and substandard quality assurance as unique LMIC-specific features which potentially add to the increasing AMR [24].

Apart from the technical challenges that may lead to AMR through inappropriate prescribing, the participating GPs identified multiple patient-level factors that may contribute to inappropriate usage of antibiotics and ultimately increase AMR, such as patient pressure on GPs to prescribe antibiotics driven by concerns regarding work and productivity loss, the tendency to self-medicate, and non-compliance with the prescribed treatment (inadequate duration, incomplete dosing). The tendency to self-medicate is of concern in LMICs, as observed in a systematic review that reported antibiotic self-medication incidence as high as 39% of all antibiotic consumption [27]. In a patient survey conducted in India, self-medicating users expressed that self-medication was harmless (66.6%) and that they would continue self-medicating (90%) and advise others to self-medicate as well (73.8%) [28]. Low socioeconomic status, lack of education, previous experience, and ease of purchasing antibiotics from pharmacies without a prescription were some of the factors contributing to self-medication, highlighting the need to educate patients about the importance of appropriate consumption of antibiotics [27].

When asked about barriers preventing them from discussing AMR with their patients, GPs cited lack of understanding and interest of patients, lack of effective educational materials, and lack of time to spend educating the patient as major reasons. In a study designed to implement an antibiotic stewardship programme in the primary care setting, patient education resulted in a 12.6% reduction in antibiotic prescription [29]. Various tools and methods such as peer education programmes and awareness surveys have been used for patient education in antibiotic stewardship programmes [30–32]. Considering the role of patient attitudes in AMR control, there is an opportunity for pharmaceutical companies to collaborate with GP associations to develop educational materials per GPs’ needs and conduct trainings to support GPs for patient education.

In addition to patient factors and the need for patient education, the survey also identified gaps in current training and the need for modern and effective training materials. Approximately 90% of the participants agreed that there is a need for antibiotic therapy-related training. Participants also believed that there are either no appropriate guidelines available or, when available, the guidelines are either not usable in the local context or are outdated. The participants also mentioned that the guidelines are typically lengthy, limiting their effective utility. Developing and providing updated and customised guidelines that cater to specific regions and consider local susceptibility data may be the most feasible route to improving prescribing practices and help decrease the risk of AMR. Recently, WHO has released the WHO AWaRe (Access, Watch, Reserve) antibiotic book to provide guidance on the appropriate use of antibiotics. This resource can aide in creating quick reference guides for GPs [33,34]. Nearly 74% of the participating GPs indicated that they would like to use quick reference guides. Quick reference materials that can be disseminated through smartphones could be a possible solution to fill the training gaps. Studies have shown that the use of smartphones for various tasks such as training, information dissemination, and time management is effective in clinical decision-making at the point of care [35,36]. Specific and regular training and continued medical education initiatives regarding appropriate antibiotic prescription and AMR for GPs were identified as unmet needs that would help improve antibiotic prescription practices in LMICs. Training and education is only one aspect to support appropriate antibiotic prescribing, with a larger need for a comprehensive support system that includes availability of quick and cost-effective laboratory tests, access to the right antibiotics, access to current susceptibility data, updated and simple local guidelines, awareness among the general public about appropriate antibiotic usage and AMR, and the right healthcare infrastructure to support appropriate antibiotic prescribing and help reduce AMR in outpatient primary care.

This study has certain limitations that should be acknowledged. Firstly, as the prescribing practices differ from country to country, the findings of our study may not be generalisable to countries beyond those included in the study. Secondly, the response rate was low, and it is unclear whether the GPs who responded to the survey invitation were particularly motivated by the subject topic compared to their peers who did not respond and may have different knowledge levels, attitudes, and practices. Additionally, we assumed that all the invited participants will have had access to appropriate devices and internet connection to be able to effectively complete the survey. However, low response rates are a known challenge in survey-based studies conducted among GPs [37]. Furthermore, as this was a blinded survey administered by a third party, the participants were not aware of the sponsor’s identity. This may have resulted in lower participation, but more unbiased responses. Finally, the opinion of 1000 GPs from nine countries may not be representative of all the attitudes and practices of all the GPs practicing in those countries or regions.

To the best of our knowledge, this is one of the largest studies assessing attitudes and antibiotic prescribing practices and AMR in 1000 + GPs across nine countries. The countries selected for this study belong to various economic categories (high-income as well as LMICs), providing diversity to the sample. Findings from this study indicate that there is an unmet need to develop effective training materials for educating patients and physicians. Furthermore, with diverse data from 9 countries with different economic contexts, it may be an interesting future analysis to assess how attitudes and practices in LMICs differ from those in higher income countries. Considering the constantly evolving landscape of AMR and related guidelines, relevant training should be offered frequently, as identified by the survey participants. The findings may also have implications for other healthcare providers, such as pharmacists, underlining the need to address practices such as self-medication.

## Conclusion

This study identifies that largely, the GPs are aware of the risks of AMR and prescribe antibiotics in appropriate settings and with presumptive diagnosis of bacterial infection. Several challenges in terms of appropriate antibiotic prescribing were noted among the surveyed GPs, along with other factors such as self‑medication practices by patients, lack of availability of crucial data and guidelines, and limited availability of diagnostic laboratories, which contribute to the increase in AMR. The participating GPs identified an unmet need for regular training programmes for physicians and, more broadly, a need for patient education. However, training is only one part of the solution, and further areas such as availability of quick and cost-effective laboratory tests, access to appropriate antibiotics, access to current susceptibility data, updated and simple local guidelines, awareness among the public about appropriate antibiotic usage and AMR, and the right healthcare infrastructure are needed to support appropriate antibiotic prescribing and help reduce AMR in outpatient primary care.

## Supporting information

S1 Table Table A: Sample Size Distribution, Table B: Understanding Antibiotic prescribing - Knowledge, Table C: Understanding Antibiotic prescribing – Attitudes, Table D: Understanding Antibiotic prescribing – Practices. (DOCX)

S1 Data Raw data. (XLSX)

S1 ICF Questionnaire Original ICF and Questionnaire used for the physician survey. (DOCX)

S1 Inclusivity Questionnaire Plos Global Public Health Inclusivity in global research questionnaire. (DOCX)
