# Supplementary material for: Attitudes and practices for antibiotic prescription and antimicrobial resistance among general physicians -Findings from a multi-country survey
Source: PLOS Glob Public Health. 2025 May 7;5(5):e0004558. doi: 10.1371/journal.pgph.0004558 (PMC12057924; doi:10.1371/journal.pgph.0004558)
Supplement: S1 Table — (DOCX) [file pgph.0004558.s001.docx]

Table A: Sample Size Distribution

| **Overall KAP response distribution* %** | 50 | 60 | 70 | 75 | 80 | 85 | 90 |
| --- | --- | --- | --- | --- | --- | --- | --- |
| Sample size | Margin of Error (± %) with 95% confidence interval | | | | | | |
| 700 | 3.70 | 3.63 | 3.39 | 3.21 | 2.96 | 2.64 | 2.22 |
| 800 | 3.46 | 3.39 | 3.17 | 3.00 | 2.77 | 2.47 | 2.08 |
| 900 | 3.27 | 3.20 | 2.99 | 2.83 | 2.61 | 2.33 | 1.96 |
| 1000 | 3.10 | 3.04 | 2.84 | 2.68 | 2.48 | 2.21 | 1.86 |
| 1100 | 2.95 | 2.89 | 2.71 | 2.56 | 2.36 | 2.11 | 1.77 |
| 1200 | 2.83 | 2.77 | 2.59 | 2.45 | 2.26 | 2.02 | 1.70 |

.

Table B: Understanding Antibiotic prescribing - Knowledge

| **Q. What is your main source of information/knowledge for prescribing antibiotics? (N=1008)** | | | | | |
| --- | --- | --- | --- | --- | --- |
| **Sources** | | | | **Weighted score** | **Rank** |
| Clinical Guidelines | | | | 2999 | 1 |
| Pharmaceutical promotion/Medical representative visits | | | | 2380 | 2 |
| Pharmaceutical Webinars/CME | | | | 1911 | 3 |
| Publications/ Scientific Papers | | | | 1848 | 4 |
| Personal experience | | | | 1720 | 5 |
| Reference Textbooks | | | | 1363 | 6 |
| Consultation with Colleagues/Peers | | | | 1271 | 7 |
| Online medical education portals (eg Medscape, CDC, WebMD etc) | | | | 1069 | 8 |
| Congresses (Conferences) | | | | 559 | 9 |
| **Q. Please respond with the degree of your agreement/disagreement to the following statement regarding use of antibiotics for upper respiratory tract infections. (N=1008)** | | | | | |
|  | **Strongly Agree**  **n (%)** | **Agree**  **n (%)** | **Neither agree/nor disagree**  **n (%)** | **Disagree**  **n (%)** | **Strongly Disagree**  **n (%)** |
| Antibiotic resistance is a concern in my region/country. | 172 (17.1) | 454 (45) | 291 (28.9) | 87 (8.6) | 4 (0.4) |
| Prescription of antibiotics in primary care can contribute to antibiotic resistance. | 108 (10.7) | 525 (52.1) | 354 (35.1) | 17 (1.7) | 4 (0.4) |
| Most URTIs are caused by viruses. | 144 (14.3) | 324 (32.1) | 391 (38.8) | 144 (14.3) | 5 (0.5) |
| Antibiotics are helpful in treating URTIs. | 103 (10.2) | 307 (30.5) | 270 (26.8) | 298 (29.6) | 30 (3) |
| Antibiotics reduce the duration of URTIs. | 99 (9.8) | 283 (28.1) | 296 (29.4) | 302 (30) | 28 (2.8) |
| Antibiotics can reduce the occurrence of complications of URTIs. | 105 (10.4) | 512 (50.8) | 329 (32.6) | 53 (5.3) | 9 (0.9) |
| Patients/Caregivers demand for antibiotics contributes to the  overuse of antibiotics in the community. | 117 (11.6) | 488 (48.4) | 315 (31.3) | 73 (7.2) | 15 (1.5) |

Table C: Understanding Antibiotic prescribing – Attitudes

| **Sources** | **Weighted score** | **Rank** |
| --- | --- | --- |
| **Q. Apart from the cause of infection, what is the major factor influencing your choice of antibiotics?** |  |  |
| Efficacy/Effectiveness/Susceptibility/Antimicrobial Resistance | 2614 | 1 |
| Tolerance of therapy by patients (Adverse events/Side effects) | 2314 | 2 |
| Clinical severity/comorbidities | 2141 | 3 |
| Scientific society/Guideline recommendations | 2034 | 4 |
| Convenient dose and duration | 1574 | 5 |
| Previous experience with an antibiotic | 1485 | 6 |
| Local availability of antibiotics | 1154 | 7 |
| Antibiotic brand | 981 | 8 |
| Cost | 823 | 9 |
| **Q. In which subset of patients do you find it most challenging to choose the right antibiotic?** | | |
| Pregnancy | 3132 | 1 |
| Patients with comorbidities | 3086 | 2 |
| Patients with recurrent infections | 2603 | 3 |
| Previous exposure to antibiotics in the last 1 month | 2493 | 4 |
| Children | 2089 | 5 |
| Elderly | 1446 | 6 |
| Adult | 271 | 7 |
| **In which of the following community-acquired respiratory tract infections, do you find it most challenging to select the right antibiotic?** | | |
| Acute exacerbation of chronic obstructive pulmonary disease (AECOPD) | 3786 | 1 |
| Community acquired pneumonia (CAP) | 3688 | 2 |
| Acute bacterial rhinosinusitis (ABRS)/Acute sinusitis | 2834 | 3 |
| Acute otitis media (AOM) | 2481 | 4 |
| Acute tonsillitis/ Acute pharyngitis | 2331 | 5 |
| **Q. Which information is most critical for you to distinguish between viral and bacterial infection in your practice?** | | |
| Clinical symptoms (eg Sputum/discharge, breathlessness, etc.) | 3797 | 1 |
| Clinical signs (e.g., Raised Temperature, dyspnea, etc.) | 3292 | 2 |
| Clinical severity | 2988 | 3 |
| Medical history (previous infections, comorbidities) | 2676 | 4 |
| Diagnostics (Imaging, Blood tests, Microbiology testing) | 2367 | 5 |
| **Q. What, in your opinion, are the main factors that drive the use of broad-spectrum antibiotics?** | | |
| To provide patients with an efficacious treatment as compared to narrow spectrum antibiotics | 3830 | 1 |
| Concerns about antimicrobial resistance | 3032 | 2 |
| To distinguish from other peers (i.e., increases physician acceptability by patients) | 2869 | 3 |
| To ensure complete eradication of a range of possible causative bacteria. | 2715 | 4 |
| Uncertainty regarding causative organisms & their susceptibility | 2674 | 5 |
| **Q. Please identify the limiting factors which may restrict appropriate antibiotic prescription, if updated local guideline; latest local antibiotic susceptibility data; and other relevant data on efficacy and appropriate prescribing are readily available?** | | |
| Data and or guidelines are not simple to understand or to use | 3756 | 1 |
| Reliance on clinical expertise/past-experience | 3436 | 2 |
| Patient/caregiver pressure to prescribe antibiotic | 3238 | 3 |
| Fear of losing patient to other physicians | 2352 | 4 |
| Lack of time | 2338 | 5 |
| **Q. What are the challenges preventing the use of guidelines to guide appropriate antibiotic selection?** | | |
| Outdated/Irrelevant guidelines | 4476 | 1 |
| Lack of easy to refer/practical guidelines | 4189 | 2 |
| Non availability of Local guidelines | 3800 | 3 |
| Rely on peer recommendations and or past experience | 3205 | 4 |
| Not aware about guidelines | 2765 | 5 |
| Lack of time | 2733 | 6 |
| **Q. Which of these do you feel are the major reasons antibiotics are prescribed without strong diagnostic evidence of bacterial infection?** | | |
| Concerns about poor or non-recovery/complications in the absence of antibiotic treatment | 2092 | 1 |
| Because further diagnostic investigations are too expensive or unavailable | 2003 | 2 |
| If the patient wants to get back to work quickly | 1914 | 3 |
| To be on the safe side (‘just in case’) | 1908 | 4 |
| The patient may not come for follow up visit concerns about patient’s not returning for the next visit | 1828 | 5 |
| If the patient demands an antibiotic (patient/caregiver expectations) | 1813 | 6 |
| Avoid imposing extra cost of another visit. | 1505 | 7 |
| Weekend or out-of- normal working hours consultation and the course of the disease is difficult to predict | 1172 | 8 |
| **Q. According to you, what are the major factors contributing to antibiotic resistance?** | | |
| Patients do not complete course of treatment | 4347 | 1 |
| Antibiotics are not prescribed in a way which would eradicate the bacterium which causes the infection | 3718 | 2 |
| Self-medication by the patient | 3414 | 3 |
| Over-prescription and consumption of antibiotics | 3352 | 4 |
| Antibiotics given at a low dose | 3313 | 5 |
| Antibiotics are of poor quality | 3024 | 6 |
| **Q. What are the barriers to discussing antimicrobial resistance (AMR) with patients when prescribing.** | | |
| Patient’s limited knowledge about the subject | 1449 | 1 |
| Unavailability of simple educational materials for patients | 1187 | 2 |
| Concern that it will unsettle the patient | 1179 | 3 |
| Lack of patient’s interest | 882 | 4 |
| It’s not important to discuss with patient | 879 | 5 |
| Lack of time | 472 | 6 |
| **Q. What intervention/activities would be most useful to you for supporting appropriate antibiotic selection?** | | |
| Frequent trainings by key specialists (eg Infectious diseases specialists, Chest Physicians, ENT specialists etc) | 4145 | 1 |
| Dedicated CMEs/Webinars/meetings | 3913 | 2 |
| Updated and regular microbiology lab reports about bacteria & Antibiotic susceptibility | 3730 | 3 |
| Website/mobile application with updated information | 3404 | 4 |
| Practical local guidelines | 3315 | 5 |
| Peer to Peer network (through independent education portals/Associations) | 2661 | 6 |
| **Q. Please choose the specific things which you would like to learn about antibiotic therapy** | | |
| Understanding pharmacokinetic and pharmacodynamic concepts for antibiotics | 2711 | 1 |
| Efficacy & safety of commonly used antibiotics | 2306 | 2 |
| Clinical criteria to distinguish between viral and bacterial infections | 1794 | 3 |
| Optimal/Recommended Dose and duration | 1698 | 4 |
| How to choose the right antibiotic for specific infections | 1301 | 5 |
| Concise summary of local/international guidelines | 1198 | 6 |
| How to understand and utilize susceptibility data | 1126 | 7 |
| Antibiotic prescription in special populations (elderly, pregnancy, comorbidities) | 889 | 8 |
| Antimicrobial stewardship in outpatient setting | 709 | 9 |
| Quality of antibiotics | 510 | 10 |
| When to refer to specialists/hospital | 447 | 11 |
| Choosing right antibiotic, dose and duration in children | 431 | 12 |

| **Q. What frequency of training/educational meetings on antibiotics would be ideal?** | | |
| --- | --- | --- |
| Frequency | n | % |
| 6 monthly | 497 | 49.3% |
| Quarterly | 333 | 33% |
| Annually | 178 | 17.7% |

| **Q. Please rate how difficult it is to select the correct/right antibiotic** | | | | | |
| --- | --- | --- | --- | --- | --- |
|  | **Extremely Easy**  **n (%)** | **Easy**  **n (%)** | **Neither easy nor difficult**  **n (%)** | **Difficult**  **n (%)** | **Extremely Difficult**  **n (%)** |
| Children (< 18 years) | 59 (5.9) | 231 (22.9) | 334 (33.1) | 338 (33.5) | 46 (4.6) |
| Adults (18 to 60 years) | 65 (6.4) | 365 (36.2) | 528 (52.4) | 43 (4.3) | 7 (0.7) |
| Elderly (>60 years) | 44 (4.4) | 191 (18.9) | 577 (57.2) | 166 (16.5) | 30 (3) |
| **Q. Which are the major challenges which make it hard for you to choose the right antibiotic**  **for empiric (initial) treatment?** | | | | | |
|  | **Strongly Agree**  **n (%)** | **Agree**  **n (%)** | **Neither agree/nor disagree**  **n (%)** | **Disagree**  **n (%)** | **Strongly Disagree**  **n (%)** |
| Difficulty in distinguishing bacterial from viral infections | 112 (11.1) | 387 (38.4) | 247 (24.5) | 257 (25.5) | 5 (0.5) |
| Lack of up-to date local treatment  guidelines | 99 (9.8) | 400 (39.7) | 416 (41.3) | 83 (8.2) | 10 (1) |
| Limited information about causative  organisms in my country/region | 96 (9.5) | 409 (40.6) | 396 (39.3) | 104 (10.3) | 3 (0.3) |
| Lack of latest susceptibility data | 83 (8.2) | 489 (48.5) | 360 (35.7) | 70 (6.9) | 6 (0.6) |
| Lack of local susceptibility data | 113 (11.2) | 448 (44.4) | 347 (34.4) | 92 (9.1) | 8 (0.8) |
| Challenging to correlate or apply  susceptibility data in clinical practice | 86 (8.5) | 445 (44.1) | 373 (37) | 98 (9.7) | 6 (0.6) |
| Complex treatment guidelines | 107 (10.6) | 406 (40.3) | 371 (36.8) | 116 (11.5) | 8 (0.8) |
| Lack of regular education/training  on antibiotics | 84 (8.3) | 431 (42.8) | 372 (36.9) | 116 (11.5) | 5 (0.5) |
| Limited access/availability of information on antibiotics  (indication, dose, interactions etc) | 100 (9.9) | 422 (41.9) | 338 (33.5) | 136 (13.5) | 12 (1.2) |
| Limited time to make a clinical  decision | 90 (8.9) | 390 (38.7) | 302 (30) | 218 (21.6) | 8 (0.8) |
| Lack of time to update myself  about latest information on  antibiotics & susceptibility | 102 (10.1) | 355 (35.2) | 323 (32) | 217 (21.5) | 11 (1.1) |
| Lack of availability of the  required/right antibiotics (Locally, hospital, institute, insurance list,  formulary) | 69 (6.8) | 442 (43.8) | 387 (38.4) | 97 (9.6) | 13 (1.3) |
| **Q. Please respond with degree of your agreement/disagreement to the following statements regarding prescription of antibiotics for upper respiratory tract infections.** | | | | | |
| I feel the patients expect antibiotics from GPs. | 113 (11.2) | 483 (47.9) | 270 (26.8) | 105 (10.4) | 37 (3.7) |
| I think that patients will change to another doctor if I do not prescribe antibiotics. | 83 (8.2) | 317 (31.4) | 375 (37.2) | 211 (20.9) | 22 (2.2) |
| I could reduce antibiotic prescription by more than 25% without jeopardizing the outcome of the URTIs. | 87 (8.6) | 288 (28.6) | 405 (40.2) | 224 (22.2) | 4 (0.4) |
| When requested to do so by patients, I prescribe antibiotics even if I think them unnecessary. | 74 (7.3) | 243 (24.1) | 197 (19.5) | 378 (37.5) | 116 (11.5) |
| Prescribing antibiotics is easier than providing explanations to the patients about their condition. | 93 (9.2) | 212 (21) | 230 (22.8) | 358 (35.5) | 115 (11.4) |
| **Q. Do you see the need of a training program about antibiotic therapy?** | | | | | |
|  | 303 (30.1) | 641 (63.6) | 54 (5.4) | 8 (0.8) | 2 (0.2) |

Table D: Understanding Antibiotic prescribing – Practices

| **Q. How often do you refer to antibiotic susceptibility data/resistance data in your routine clinical practice when prescribing antibiotics?** | | |
| --- | --- | --- |
| Frequency | n | % |
| Often | 499 | 49.5% |
| Sometimes | 315 | 31.3% |
| Always | 194 | 19.2% |
| Never | 0 | 0% |
| **Sources** | **Weighted score** | **Rank** |
| **Q. What resources do you use to understand antibiotic susceptibility and antimicrobial resistance to help you choose the right antibiotic?** | | |
| Guidelines (Local/International) | 3313 | 1 |
| Lab microbiology reports | 3246 | 2 |
| CME/Webinars | 2914 | 3 |
| International susceptibility data (Publications/Surveillance network) | 2913 | 4 |
| Local susceptibility data (Publications/Surveillance network) | 2734 | 5 |
| **Q. Which approach do you follow most of the time when prescribing antibiotics?** | | |
| Approach | n | % |
| Wait and watch (Delayed prescribing) | 617 | 61.2% |
| Immediate prescribing | 391 | 38.8% |
| **Q. Do you refer to guidelines to help choose an appropriate antibiotic in your routine clinical practice?** | | |
| Frequency | n | % |
| Often | 453 | 44.9% |
| Sometimes | 323 | 32% |
| Always | 227 | 22.5% |
| Never | 5 | 0.5% |
| **Q. What is your preferred mode to participate in education and training?** | | |
| Mode | n | % |
| Digital /Online meetings |  |  |
| Most preferred | 414 | 41.1% |
| Preferred | 355 | 35.2% |
| Least preferred | 239 | 23.7% |
| In-person meetings |  |  |
| Most preferred | 327 | 32.4% |
| Preferred | 283 | 28.1% |
| Least preferred | 398 | 39.5% |
| Mix of digital + In-person meetings |  |  |
| Most preferred | 267 | 26.5% |
| Preferred | 370 | 36.7% |
| Least preferred | 371 | 36.8% |
| **Q. Would you use quick reference materials/documents (ex. Pocket guides/infographics) in addition to the above meetings** | | |
| Response | n | % |
| Yes | 746 | 74% |
| No | 262 | 26% |
| **Sources** | **Weighted score** | **Rank** |
| **Q. Please select your preferred medium to access educational content/materials (such as short videos, PDFs, infographics etc)** | | |
| Through existing local physician association portals/website | 3091 | 1 |
| Website | 2559 | 2 |
| Physician social platforms (e.g. Sermo, Figure 1, Daily rounds etc) | 2397 | 3 |
| Through educational platforms which offer credit/CME points | 1927 | 4 |
| Mobile application | 1796 | 5 |
| WhatsApp– (Or similar messaging application in your country) | 1543 | 6 |
| Email | 1036 | 7 |
| You Tube channel | 771 | 8 |

| **Q. How often do you prescribe an antibiotic for patients with the following clinical presentations**  **(without special investigations or diagnostic support)?** | | | | | |
| --- | --- | --- | --- | --- | --- |
|  | Always (> 80%)  n (%) | Almost always (60-80%)  n (%) | Sometimes (40-60 %)  n (%) | Almost Never (20-40%)  n (%) | Never (<20%)  n (%) |
| **CHILDREN** |  |  |  |  |  |
| Runny nose with yellow or green mucus discharge | 109 (10.8) | 302 (30) | 385 (38.2) | 167 (16.6) | 45 (4.5) |
| Sore throat + Fever | 84 (8.3) | 433 (43) | 377 (37.4) | 88 (8.7) | 26 (2.6) |
| Fever + Coughing | 131 (13) | 359 (35.6) | 404 (40.1) | 100 (9.9) | 14 (1.4) |
| Common Cold | 101 (10) | 332 (32.9) | 356 (35.3) | 159 (15.8) | 60 (6) |
| Fever + pleuritic chest pain + breathing problems | 77 (7.6) | 368 (36.5) | 445 (44.1) | 100 (9.9) | 18 (1.8) |
| Ear pain + fever | 57 (5.7) | 293 (29.1) | 477 (47.3) | 160 (15.9) | 21 (2.1) |
| Runny nose /Facial pain/ Headache | 64 (6.3) | 345 (34.2) | 434 (43.1) | 125 (12.4) | 40 (4) |
| Breathing problem + cough + fever | 65 (6.4) | 437 (43.4) | 400 (39.7) | 83 (8.2) | 23 (2.3) |
| **ADULT** | | | | | |
| Runny nose with yellow or green mucus discharge | 91 (9) | 386 (38.3) | 371 (36.8) | 124 (12.3) | 36 (3.6) |
| Sore throat + Fever | 118 (11.7) | 441 (43.8) | 330 (32.7) | 94 (9.3) | 25 (2.5) |
| Fever + Coughing | 123 (12.2) | 402 (39.9) | 372 (36.9) | 92 (9.1) | 19 (1.9) |
| Common Cold | 115 (11.4) | 383 (38) | 305 (30.3) | 150 (14.9) | 55 (5.5) |
| Fever + pleuritic chest pain + breathing problems | 100 (9.9) | 393 (39) | 342 (33.9) | 126 (12.5) | 47 (4.7) |
| Ear pain + fever | 65 (6.4) | 325 (32.2) | 451 (44.7) | 141 (14) | 26 (2.6) |
| Runny nose /Facial pain/ Headache | 72 (7.1) | 411 (40.8) | 359 (35.6) | 131 (13) | 35 (3.5) |
| Breathing problem + cough + fever | 85 (8.4) | 434 (43.1) | 329 (32.6) | 113 (11.2) | 47 (4.7) |
