# Supplementary material for: Attitudes and practices for antibiotic prescription and antimicrobial resistance among general physicians -Findings from a multi-country survey
Source: PLOS Glob Public Health. 2025 May 7;5(5):e0004558. doi: 10.1371/journal.pgph.0004558 (PMC12057924; doi:10.1371/journal.pgph.0004558)
Supplement: S1 ICF Questionnaire — (DOCX) [file pgph.0004558.s003.docx]

**Knowledge, Attitudes and Practices Towards Antibiotic Use by General Practitioners to Manage Community Acquired Respiratory Tract Infections**

| **Informed Consent Form**  You are invited to take part in this research study. This study is conducted by EVERSANA, on behalf of the sponsor. The study has been reviewed and approved by an Independent Ethics Committee (IEC). IECs protect the rights, safety and well-being of people who take part in research studies. This consent form describes the study and what taking part in the study means for you. Your decision to take part in this study is voluntary. It is completely up to you. You can change your decision and leave the study at any time without giving a reason.  What is the purpose of this study?  This research study is being done to learn more about the knowledge, attitude, and practices toward antibiotic use by General Practitioners (GPs) to manage community acquired respiratory tract infections. About 1000 GPs from up to 10 countries will take part in this study. The survey should take about 30 minutes.  What do you need to do in this study?  If you choose to take part in this study, you will need to provide consent by clicking on continue at the end of this form. If you agree to join this study then you also need to answer a set of questions related to the knowledge, attitude, and practices toward use of antibiotics in community acquired respiratory tract infections.  What potential benefits can you expect?  You will not receive any direct benefit by participating in this study.  What will happen to your personal information (data)?  Your personal information will not be collected as part of this survey. To protect your identity, you will be assigned a case identification number and no personal information will be collected as part of the survey.  Can you leave the study? What happens if you leave the study?  You can leave the study at any time without giving any reason. If you leave the survey in between, your responses collected while you were in the study will remain as part of the study and will be used for analysis of the study results.  You may choose not to participate, and you may withdraw at any time during the research project. You will NOT be penalized in any way should you choose not to participate or to withdraw.  Will you be paid for taking part in the study?  Should you choose to participate in the survey, you will be awarded remuneration upon completion of the survey based on applicable jurisdiction in your country.  Do you have to pay anything to take part in the study?  You do not have to pay to take part in this study.  How your data may be used?  Why will your data be collected?  The sponsor conducting the research will use the responses provided by you to the questions to:   - Carry out this study and meet the study purpose. - Understand results of this study. - Publish results of the study. If we do this, your name will not appear in any publication.   How is your personal information protected?  All appropriate measures will be taken to protect your personal information. These measures will comply with data protection and privacy laws that apply.  As part of this effort, your identity will not be revealed in any publications that result from this study. The information in the study records will be kept strictly confidential. To protect your identity, you will be assigned a case identification number. Individual data will be stored securely and will be made available only to the people conducting the study i.e., people working on behalf of sponsor and ethics committee. No reference will be made in oral or written reports that could link you to the study.  Consent statement  **I understand the purpose of this study and the content of this form. I had enough time to decide whether I want to take part in the study. I am aware that I can change my mind and leave the study at any time without giving a reason.**  If you decide to participate, please proceed further and click on **Continue.**  If you decide not to participate, please **Exit**. |
| --- |

**Section I: Demographics**

| **S1** | Age | ______ years |
| --- | --- | --- |
| **S2** | Gender |    |
| **S3** | Country of Practice | India  Pakistan  Algeria  Vietnam  Morocco  Saudi Arabia/Kuwait  Egypt  UAE  Thailand |
| **S4** | Highest Qualification |  |
| **S5** | How long have you been a medical practitioner? | ______ years |
| **S6** | How many patients do you see with community-acquired bacterial infections (for example bacterial respiratory tract infections, skin & soft tissue infections, etc) in a typical month? | ___ adult patients per month  ___ child patients per month |
| [Terminate if S5 < 5 years or Sum of S6<20] | | |
| **S7** | Please indicate the setting in which you practice (i.e., where do you see most of your patients) | **Government hospital**  **Government OPD (Outpatient Clinic)**  **Private Hospital**  **Private Outpatient facility/Consulting rooms (Outpatient Clinic)**  **Primary health care clinic (Outpatient**) |
| **S8** | Please indicate the type of geographical area/location you practice. | **Rural**  **Semi-Rural**  **Urban**  **Semi-urban** |
| **S9** | Payment mode at your clinic/hospital. Tick all that apply | **Private Insurance**  **Public Insurance**  **Out of Pocket Payment** |

**Section:2 Understanding Antibiotic prescribing - Knowledge, Attitudes, Practices**

**Please note that all questions pertain to oral antibiotic use only.**

| **1** | **What is your main source of information/knowledge for prescribing antibiotics?** | **Select the top 5 and rank in the order of priority from 1 to 5**  i. Clinical Guidelines    ii. Reference Textbooks    iii. Publications/ Scientific Papers    iv. Personal experience    v. Consultation with Colleagues/Peers  vi. Pharmaceutical promotion/Medical representative visits    vii. Pharmaceutical Webinars/CME    viii. Online medical education portals (eg Medscape, CDC, WebMD etc)      ix. Congresses (Conferences)  x. Others (Please mention) |
| --- | --- | --- |
| 2 | **Apart from the cause of infection, what is the major factor influencing your choice of antibiotics?** | **Select the top 5 and rank in the order of priority from 1 to 5**    i. Cost  ii. Clinical severity/comorbidities    iii. Convenient dose and duration    iv. Tolerance of therapy by patients (Adverse events/Side effects)    v. Local availability of antibiotics    vi. Efficacy/Effectiveness/Susceptibility/Antimicrobial Resistance    vii. Scientific society/Guideline recommendations  viii. Previous experience with an antibiotic    ix. Antibiotic brand    x. Others (Please mention) |
| 3 | **How often do you prescribe an antibiotic for patients with the following clinical presentations (without special investigations or diagnostic support)?** | **CHILDREN**   \| **Infection /condition** \| **Always**  **(> 80%)** \| **Almost always**  **(60-80%)** \| **Sometimes**  **(40-60 %)** \| **Almost Never**  **(20-40%)** \| **Never (<20%)** \| \| --- \| --- \| --- \| --- \| --- \| --- \| \| Runny nose with yellow or green mucus discharge \| **** \| **** \| **** \| **** \| **** \| \| Sore throat + Fever \| **** \| **** \| **** \| **** \| **** \| \| Fever + Coughing \| **** \| **** \| **** \| **** \| **** \| \| Common Cold \| **** \| **** \| **** \| **** \| **** \| \| Fever + pleuritic chest pain + breathing problems \| **** \| **** \| **** \| **** \| **** \| \| Ear pain + fever \| **** \| **** \| **** \| **** \| **** \| \| Runny nose /Facial pain/ Headache \| **** \| **** \| **** \| **** \| **** \| \| Breathing problem + cough + fever \| **** \| **** \| **** \| **** \| **** \|       **ADULTS**   \| **Infection /condition** \| **Always (> 80%)** \| **Almost always**  **(60-80%)** \| **Sometimes**  **(40-60 %)** \| **Almost Never**  **(20-40%)** \| **Never (<20%)** \| \| --- \| --- \| --- \| --- \| --- \| --- \| \| Runny nose with yellow or green mucus discharge \| **** \| **** \| **** \| **** \| **** \| \| Sore throat + Fever \| **** \| **** \| **** \| **** \| **** \| \| Fever + Coughing \| **** \| **** \| **** \| **** \| **** \| \| Common Cold \| **** \| **** \| **** \| **** \| **** \| \| Fever + pleuritic chest pain + breathing problems \| **** \| **** \| **** \| **** \| **** \| \| Ear pain + fever \| **** \| **** \| **** \| **** \| **** \| \| Runny nose /Facial pain/ Headache \| **** \| **** \| **** \| **** \| **** \| \| Breathing problem + cough + fever \| **** \| **** \| **** \| **** \| **** \| |
| **4** | **Please rate how difficult it is to select the correct/right antibiotic.** | \| **Age Group** \| **Extremely Easy** \| **Easy** \| **Neither easy nor difficult** \| **Difficult** \| **Extremely Difficult** \| \| --- \| --- \| --- \| --- \| --- \| --- \| \| Children  (< 18 years) \| **** \| **** \| **** \| **** \| **** \| \| Adults  (18 to 60 years) \| **** \| **** \| **** \| **** \| **** \| \| Elderly  (>60 years) \| **** \| **** \| **** \| **** \| **** \| |
| **5** | **In which subset of patients do you find it most challenging to choose the right antibiotic?** | **Select the top 5 and rank in the order of priority from 1 to 5**    i. Children    ii. Elderly    iii. Pregnancy    iv. Patients with comorbidities    v. Patients with recurrent infections    vi. Previous exposure to antibiotics in the last 1 month  vii. Adult |
| **6** | **In which of the following community-acquired respiratory tract infections, do you find it most challenging to select the right antibiotic?** | **Please rank from 1 to 5, with 1 being the most challenging and 5 being the least challenging**    i. Community acquired pneumonia (CAP)    ii. Acute otitis media (AOM)  iii. Acute bacterial rhinosinusitis (ABRS)/Acute sinusitis    iv. Acute exacerbation of chronic obstructive pulmonary disease (AECOPD)    v. Acute tonsillitis/ Acute pharyngitis |
| **7** | **Which are the major challenges which make it hard for you to choose the right antibiotic for empiric (initial) treatment?** | \|  \| Strongly  Agree \| Agree \| Neither Agree /  Nor disagree \| Disagree \| Strongly Disagree \| \| --- \| --- \| --- \| --- \| --- \| --- \| \| Difficulty in distinguishing bacterial from viral infections \| **** \| **** \| **** \|  \|  \| \| Lack of up-to date local treatment guidelines \|  \|  \|  \|  \|  \| \| Limited information about causative organisms in my country/region \|  \|  \|  \|  \|  \| \| Lack of latest susceptibility data \|  \|  \|  \|  \|  \| \| Lack of local susceptibility data \|  \|  \|  \|  \|  \| \| Challenging to corelate or apply susceptibility data in clinical practice \|  \|  \|  \|  \|  \| \| Complex treatment guidelines \|  \|  \|  \|  \|  \| \| Lack of regular education/training on antibiotics \|  \|  \|  \|  \|  \| \| Limited access/availability of information on antibiotics (indication, dose, interactions etc) \|  \|  \|  \|  \|  \| \| Limited time to make a clinical decision \|  \|  \|  \|  \|  \| \| Lack of time to update myself about latest information on antibiotics & susceptibility \|  \|  \|  \|  \|  \| \| Lack of availability of the required/right antibiotics (Locally, Hospital, institute, insurance list, formulary) \|  \|  \|  \|  \|  \| |
| **8** | **Which information is most critical for you to distinguish between viral and bacterial infection in your practice?** | **Please rank from 1 to 5, with 1 being the most critical/useful and 5 being the least critical/useful**    i. Medical history (previous infections, comorbidities)    ii. Clinical severity    iii. Clinical symptoms (eg Sputum/discharge, breathlessness, etc.)    iv. Clinical signs (e.g., Raised Temperature, dyspnea, etc.)  v. Diagnostics (Imaging, Blood tests, Microbiology testing)    vi. Others (Please specify) |
| **9** | **How often do you refer to antibiotic susceptibility data/resistance data in your routine clinical practice when prescribing antibiotics?** |  |
| **10** | **What resources do you use to understand antibiotic susceptibility and antimicrobial resistance to help you choose the right antibiotic?** | **Please rank from 1 to 5, with 1 being the most important/most used and 5 being the least important/least used**    i. Guidelines (Local/International)    ii. Local susceptibility data (Publications/Surveillance network)    iii. Lab microbiology reports    iv. International susceptibility data (Publications/ Surveillance network)  v. CME/Webinars    vi. Others (Please mention) |
| **11** | **Please respond with the degree of your agreement/disagreement to the following statements regarding use of antibiotics for upper respiratory tract infections.** | \|  \| Strongly Agree \| Agree \| Neither  agree/  Nor  Disagree \| Disagree \| Strongly Disagree \| \| --- \| --- \| --- \| --- \| --- \| --- \| \| Antibiotic resistance is a concern in my region/country. \|  \|  \|  \|  \|  \| \| Prescription of antibiotics in primary care can contribute to antibiotic resistance. \|  \|  \|  \|  \|  \| \| Most URTIs are caused by viruses. \|  \|  \|  \|  \|  \| \| Antibiotics are helpful in treating URTIs. \|  \|  \|  \|  \|  \| \| Antibiotics reduce the duration of URTIs. \|  \|  \|  \|  \|  \| \| Antibiotics can reduce the occurrence of complications of URTIs. \|  \|  \|  \|  \|  \| \| Patients/Caregivers demand for antibiotics contributes to the overuse of antibiotics in the community. \|  \|  \|  \|  \|  \| |
| **12** | **Please respond with degree of your agreement/disagreement to the following statements regarding prescription of antibiotics for upper respiratory tract infections.** | \|  \| Strongly Agree \| Agree \| Neither agree/  Nor Disagree \| Disagree \| Strongly Disagree \| \| --- \| --- \| --- \| --- \| --- \| --- \| \| I feel the patients expect antibiotics from GPs. \|  \|  \|  \|  \|  \| \| I think that patients will change to another doctor if I do not prescribe antibiotics. \|  \|  \|  \|  \|  \| \| I could reduce antibiotic prescription by more than 25% without jeopardizing the outcome of the URTIs. \|  \|  \|  \|  \|  \| \| When requested to do so by patients, I prescribe antibiotics even if I think them unnecessary. \|  \|  \|  \|  \|  \| \| Prescribing antibiotics is easier than providing explanations to the patients about their condition. \|  \|  \|  \|  \|  \| |
| **13** | **Which approach do you follow most of the time when prescribing antibiotics?** |  |
| **14** | **What, in your opinion, are the main factors that drive the use of broad-spectrum antibiotics?** | **Please rank from 1 to 5, with 1 being the most important and 5 being the least important**    i. Concerns about antimicrobial resistance    ii. Uncertainty regarding causative organisms & their susceptibility  iii. To distinguish from other peers (i.e., increases physician    acceptability by patients)    iv. To provide patients with an efficacious treatment as compared  to narrow spectrum antibiotics    v. To ensure complete eradication of a range of possible causative  bacteria. |

**Section:3 Understanding reasons for behavior/Intervention Questions**

| **15** | **Please identify the limiting factors which may restrict appropriate antibiotic prescription, if updated local guideline; latest local antibiotic susceptibility data; and other relevant data on efficacy and appropriate prescribing are readily available?** | **Please rank from 1 to 5, with 1 being the most prominent challenge to 5 being the least likely reason**  i. Data and or guidelines are not simple to understand    or to use    ii. Lack of time  iii. Reliance on clinical expertise/past-experience      iv. Patient/caregiver pressure to prescribe antibiotic  v. Fear of losing patient to other physicians      vi. Any other reason (Please specify) |
| --- | --- | --- |
| **16** | **Do you see the need of a training program about antibiotic therapy?**  **(Selecting the right antibiotic for right disease/ case)** |  |
| **17** | **Do you refer to guidelines to help choose an appropriate antibiotic in your routine clinical practice?** |  |
| **18** | **What are the challenges preventing the use of guidelines to guide appropriate antibiotic selection?** | **Please rank from 1 to 6, with 1 being the most important and 6 being the least important**    i. Lack of time    ii. Non availability of Local guidelines    iii. Outdated/Irrelevant guidelines    iv. Lack of easy to refer/practical guidelines  v. Not aware about guidelines    vi. Rely on peer recommendations and or past    experience |
| **19** | **Which of these do you feel are the major reasons antibiotics are prescribed without strong diagnostic evidence of bacterial infection?** | **Select the top 5 and rank in the order of priority from 1 to 5**  i. Weekend or out-of- normal working hours consultation    and the course of the disease is difficult to predict  ii. If the patient wants to get back to work quickly    iii. If the patient demands an antibiotic (patient/    caregiver expectations)    iv. Because further diagnostic investigations are too  expensive or unavailable    v. To be on the safe side (‘just in case’)  vi. The patient may not come for follow up visit concerns    about patient’s not returning for the next visit  vii. Concerns about poor or non-recovery/complications    in the absence of antibiotic treatment    viii. Avoid imposing extra cost of another visit. |
| **20** | **According to you, what are the major factors contributing to antibiotic resistance?** | **Please rank from 1 to 6, with 1 being the most important and 6 being the least important**    i. Patients do not complete course of treatment  ii. Antibiotics are not prescribed in a way which    would eradicate the bacterium which causes the  infection    iii. Antibiotics given at a low dose    iv. Antibiotics are of poor quality    v. Over-prescription and consumption of antibiotics  vi. Self-medication by the patient    vii. Any other factors |
| **21** | **What are the barriers to discussing antimicrobial resistance (AMR) with patients when prescribing.** | **Select the top 3 and rank in the order of priority from 1 to 3:**    i. Lack of time    ii. Concern that it will unsettle the patient  iii. Lack of patient’s interest    iv. Patient’s limited knowledge about the subject    v. It’s not important to discuss with patient    vi. Unavailability of simple educational materials    for patients |
| **22** | **What intervention/activities would be most useful to you for supporting appropriate antibiotic selection?** | **Please rank from 1 to 6, with 1 being the most useful and 6 being the least useful**    i. Dedicated CMEs/Webinars/meetings    ii. Practical local guidelines  iii. Website/mobile application with updated information    iv. Frequent trainings by key specialists    (eg Infectious diseases specialists, Chest Physicians,  ENT specialists etc)  v. Updated and regular microbiology lab reports    about bacteria & Antibiotic susceptibility  vi. Peer to Peer network (through independent    education portals/Associations)    vii. Others (please specify) |
| **23** | **What is your preferred mode to participate in education and training?** | **Please rate from 1 to 3, where 1 is most preferred and 3 is least preferred.**    i. Digital /Online meetings    ii. In-person meetings    iii. Mix of digital + F2F meetings |
| **24** | **Would you use quick reference materials/documents (ex. Pocket guides/infographics) in addition to the above meetings** |  |
| **25** | **Please select your preferred medium to access educational content/materials (such as short videos, PDFs, infographics etc)** | **Select the top 5 and rank in the order of priority from 1 to 5**    i. Email    ii. Website  iii. Mobile application    iv. Through existing local physician association    portals/website  v. WhatsApp– (Or similar messaging application in    your country)    vi. Physician social platforms (e.g. Sermo, Figure 1,  Daily rounds etc)    vii. You Tube channel    viii. Through educational platforms which offer credit/  CME points    Others (Please specify) |
| **26** | **What frequency of training/educational meetings on antibiotics would be ideal?** | Others (Please specify) |
| **27** | **Please choose the specific things which you would like to learn about antibiotic therapy** | **Select the top 5 and rank in the order of priority from 1 to 5**    i. How to choose the right antibiotic for specific  infections    ii. Optimal/Recommended Dose and duration    iii. Efficacy & safety of commonly used antibiotics  iv. Understanding pharmacokinetic and    pharmacodynamic concepts for antibiotics    v. How to understand and utilize susceptibility data  vi. Concise summary of local/international guidelines    vii. Clinical criteria to distinguish between viral and    bacterial infections    viii. When to refer to specialists/hospital  ix. Antimicrobial stewardship in outpatient setting    x. Quality of antibiotics    xi. Choosing right antibiotic, dose and duration in    children    xii. Antibiotic prescription in special populations  (elderly, pregnancy, comorbidities)    xiii. Others (Please specify) |
| **28** | **Do you have any specific suggestions/interventions which would help you to choose appropriate antibiotic in your day-to-day practice?** | **Open Text** |
